# Validation of the World Falls Guidelines risk stratification algorithm. A systematic review

**DOI:** 10.1093/ageing/afag231

**Published:** 2026-08-12

**Authors:** Frederico Pieruccini-Faria, Surim Son, Kim Delbaere, Nathalie van der Velde, Stephen R Lord, Finbarr C Martin, Jesper Ryg, Maw Pin Tan, Teresa Liu-Ambrose, Diana Amaris, Cameron Hicks, Tahir Masud, Manuel Montero-Odasso

**Affiliations:** Department of Medicine, Division of Geriatric Medicine, Schulich School of Medicine & Dentistry, Western University, 1151 Richmond Street, London, Ontario N6C 3K7, Canada; Gait and Brain Lab, Parkwood Institute and Lawson Research Institute, 550 Wellington Rd, London, Ontario N6C 5J1, Canada; Department of Epidemiology and Biostatistics, Schulich School of Medicine & Dentistry, Western University, 1151 Richmond Street, London, Ontario N6C 3K7, Canada; Division of Clinical Geriatrics, Department of Neurobiology, Care Sciences and Society, Karolinska Institutet, Stockholm, Sweden; School of Health Sciences, University of New South Wales, Health Translation Hub, Randwick, New South Wales 2031, Australia; Falls, Balance and Injury Research Centre, Neuroscience Research Australia, Randwick, New South Wales 2031, Australia; Amsterdam UMC—Locatie AMC—Internal Medicine, Section of Geriatric Medicine, Amsterdam Public Health, Meibergdreef 9, Amsterdam, North Holland 1105 AZ, Netherlands; Amsterdam Public Health Research Institute, Amsterdam UMC, University of Amsterdam 1081 AZ Amsterdam, The Netherlands; Neuroscience Research Australia, UNSW, Sydney, New South Wales, Australia; Population Health Sciences, King’s College London Faculty of Life Sciences & Medicine, Addison House, Guy’s Campus Great Maze Road, London SE1 9RT, UK; Ageing and Health, Guy’s and St Thomas’ Hospitals NHS Trust, Westminster Bridge Road, London SE1 7EH, UK; Department of Clinical Medicine, University of Copenhagen, Copenhagen, Denmark; Geriatric Research and Clinical Evidence (GRACE), Department of Internal Medicine, Copenhagen University Hospital - Herlev and Gentofte Hospital, Copenhagen, Denmark; Department of Medicine, Faculty of Medicine Universiti Malaya (University of Malaya) Kuala Lumpur, Malaysia; Faculty of Medicine, The University of British Columbia, Vancouver, British Columbia, Canada; Medicine, Lawson Health Research Institute, London, Ontario, Canada; Neuroscience Research Australia, 139 Barker St, Randwick, New South Wales 2031, Australia; Healthcare for Older People, Nottingham University Hospitals NHS Trust, Queens Medical Centre Campus, Nottingham NG7 2UH, UK; Department of Medicine, Division of Geriatric Medicine, Schulich School of Medicine & Dentistry, Western University, 1151 Richmond Street, London, Ontario N6C 3K7, Canada; Gait and Brain Lab, Parkwood Institute and Lawson Research Institute, 550 Wellington Rd, London, Ontario N6C 5J1, Canada; Department of Epidemiology and Biostatistics, Schulich School of Medicine & Dentistry, Western University, 1151 Richmond Street, London, Ontario N6C 3K7, Canada

**Keywords:** falls, guidelines, falls algorithm, falls in older adults, systematic review, older people

## Abstract

**Background:**

Falls are a major cause of morbidity and mortality in older adults. The World Falls Guidelines (WFG) propose a risk stratification algorithm using fall history, three key questions (3KQ), mobility tests and clinical criteria, but it has yet to be systematically evaluated.

**Objective:**

To assess whether the WFG algorithm, in original or modified form, effectively stratifies community-dwelling older adults into low, intermediate or high risk of future falls; whether it has been prospectively validated; and whether it has been adequately operationalised.

**Methods:**

We conducted a systematic review (PROSPERO CRD420251151506) by searching MEDLINE, Embase and Google Scholar. Two reviewers independently screened studies and extracted data on cohort characteristics, algorithm adaptations, baseline risk distribution and prospective falls outcomes over ≥6 months. Given the heterogeneity of the studies included, quantitative meta-analysis was not conducted.

**Results:**

Eight cohort studies (*n* = 25 027; mean age range 61–82 years) met the inclusion criteria. No study implemented the WFG algorithm as published; two used minor modifications and six had major adaptations due to dataset limitations. Common modifications included rephrasing the 3KQ, replacing ‘subjective unsteadiness’ question with objective measures, applying mobility test universally, adjusting mobility tests thresholds and omitting fall severity definition components. Across studies, 7.9%–82.6% of participants were classified as low risk, 0.4%–18.3% as intermediate risk and 10.5%–88.7% as high risk. High-risk classification showed modest sensitivity (26.5%–52.3%) and moderate-to-high specificity (34.2%–88.9%) for future falls (1–2 years), with an overall accuracy of 61.2%–74.2%. High-risk groups consistently had a two- to three-fold higher incidence of falls than low-risk groups, while intermediate-risk groups only showed a slightly elevated risk. Use of 3KQ alongside fall history and universal mobility testing improved discrimination and increased the proportion in the intermediate-risk category.

**Conclusion:**

The operationalised versions of the WFG algorithm identify older adults at high falls risk with high specificity but limited sensitivity. Proposed pragmatic modifications improve stratification accuracy for intermediate risk group but require prospective validation.

## Key Points

- The World Falls Guidelines (WFG) algorithm for fall risk stratification in older adults requires validation.
- Across eight cohort studies (*n* = 25 027), high-risk groups were identified with good specificity but modest sensitivity.
- Intermediate-risk groups were poorly classified and no study used the algorithm exactly as published.
- Universal mobility testing with more flexible cut-offs using the three key questions improved sensitivity and detection of intermediate risk.
- The WFG algorithm reliably identifies high-risk older adults; modifications may enhance risk stratification, but prospective validation is needed

## Introduction

Older adults face a high risk of harm from falls [1], including fatal and non-fatal injuries, disability, hospitalisation and institutionalisation. Beyond physical injury, falls are associated with concerns about falling, loss of confidence and reduced activity which compound functional decline [2]. Despite evidence-based risk-reducing interventions such as balance-challenging and functional exercises [3–5], falls remain a substantial and growing public health challenge [6]. In North America alone, fall-related costs are estimated at $56 billion annually [7–9], particularly among adults 80 years and older, accounting for a substantial proportion of this burden due to their increased risk of sustaining fall-related injuries requiring medical care. Despite this substantial burden, the prevalence of falls among community-dwelling adults aged 65 and older has remained largely unchanged over the past three decades [10, 11]. These trends have intensified global efforts to address falls prevention.

**Figure 1:**
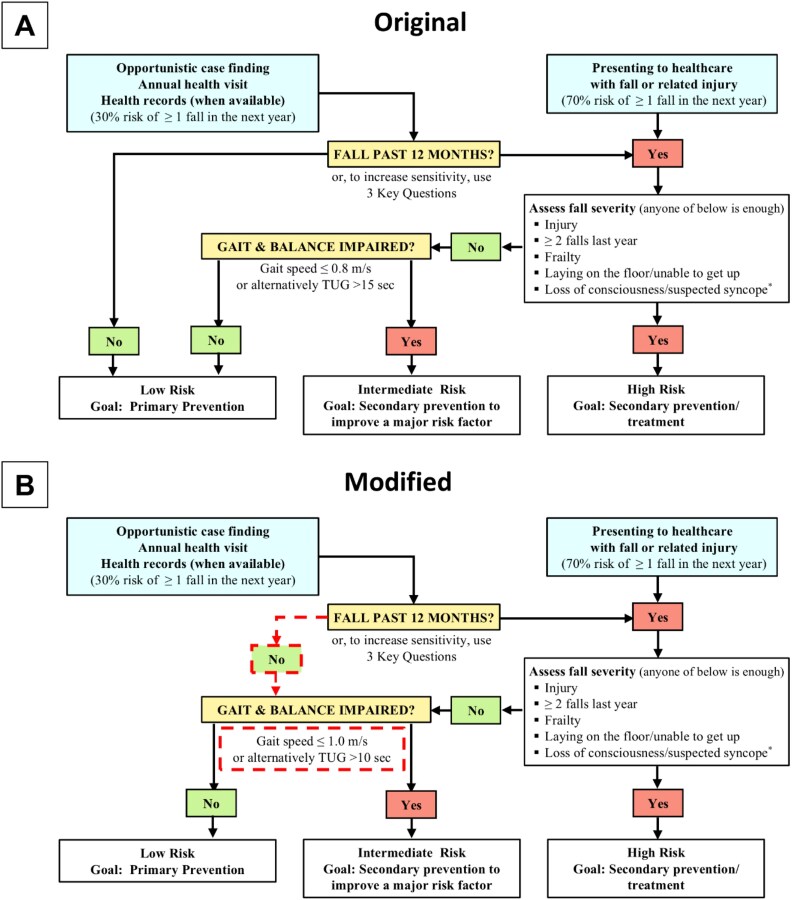
(A) Original World Falls Guidelines algorithm proposed by experts; (B) World Falls Guidelines algorithm showing proposed modifications (red box, dashed lines) informed by the systematic review with a gait speed cut-off of 1 m/s whereas TUG cut-off time of 10s.

The World Falls Guidelines (WFG) [12, 13] have been widely cited and tested or implemented in several countries [14, 15], highlighting their influence on research and clinical practice. At the primary care level, to facilitate implementation, the WFG introduced a risk stratification algorithm categorising older adults as ‘low’, ‘intermediate’ or ‘high’ risk. Developed through evidence synthesis and expert consensus, the algorithm (Figure 1A) begins with a single screening question on falls in the past 12 months [16–19]. Screening sensitivity is enhanced through three key questions (3KQ) assessing recent falls, unsteadiness while standing or walking and concerns about falling [20]. Individuals with negative responses to all 3KQ are classified as low risk. Those with a positive response [20] proceed to a fall risk severity assessment, which includes questions on injurious or recurrent falls, frailty, prolonged inability to rise after a fall and suspected loss of consciousness. Any positive response to these additional questions results in a classification of high-risk, for which a comprehensive multifactorial falls assessment is recommended. Individuals with negative responses to each component proceed to a mobility assessment using gait speed or the timed up and go (TUG) test. Abnormal mobility results [21] indicate intermediate risk, for which tailored exercise programs are offered.

The WFG algorithm is intended to guide clinical decision-making and allocate fall-prevention resources by enabling accurate risk stratification across populations. While it was developed based on available evidence and expert consensus, empirical validation is required to confirm its effectiveness for risk stratification and prediction. A systematic evaluation of its operationalisation and performance metrics is essential to determine its practical applicability. Our objective was to synthesise the evidence on the algorithm’s validity for stratifying community-dwelling older adults into low, intermediate and high risk of future falls and to assess whether it has been prospectively validated and adequately operationalised. Specifically, we aimed to address three questions: (i) Does the WFG algorithm accurately stratify fall risk among community-dwelling older adults? (ii) Has it been prospectively validated? (iii) What modifications have been proposed to improve its accuracy?

## Methods

This systematic review (PROSPERO CRD420251151506) follows the Preferred Reporting Items for Systematic reviews and Meta-Analyses (PRISMA-DTA) statement for reporting (Supplementary Appendix 1) [22].

### Eligibility criteria

Publications were included if they met the following criteria: (i) participants had a mean age ≥60 years; (ii) the WFG algorithm was used to stratify falls risk; (iii) the proportion of participants in each risk group and falls incidence by risk group were reported; and (iv) they had at least 6 months of follow-up. No language restrictions were applied. Studies were excluded if they did not operationalise or validate the WFG algorithm, used randomised clinical trial or interventional designs (which may influence falls incidence), were not peer-reviewed, or comprised conference abstracts, study protocols or commentaries. Operationalisation of the WFG algorithm was defined as application of all recommended steps for risk stratification, using either the specified tests or acceptable proxies. Where one or more components were missing, the co-lead and senior authors jointly determined eligibility based on whether the omission materially compromised the validity of risk stratification.

### Search strategy and study selection

**Figure 2:**
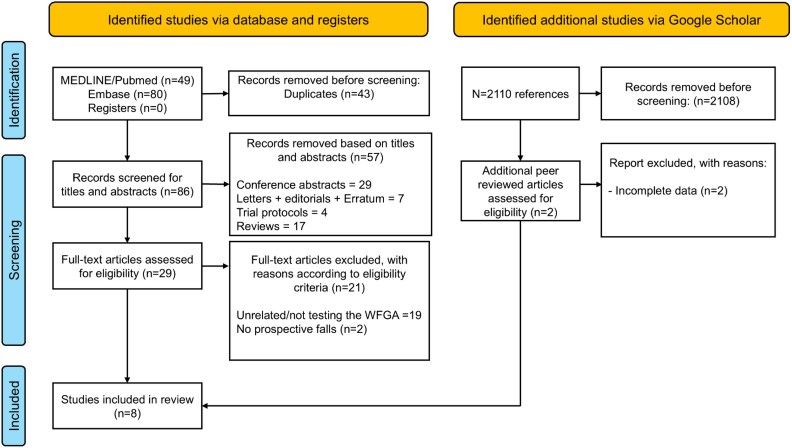
PRISMA flowchart for identification, screening, and inclusion of searched studies in the systematic review.

Search was conducted in the MEDLINE/PubMed and Embase databases from September 2022 to 10 November 2025; and updated on 27 April 2026 using the Ovid platform to maximise retrieval, including the terms ‘world falls guidelines’ or ‘world guidelines’ and ‘falls’ or ‘accidental falls’ (see Supplementary Appendix 2). The same search strategy was also conducted for the manual citation search in Google Scholar (Figure 2). Two reviewers (F.P.-F. and S.S.) independently screened studies by title and abstract, followed by full text review. Disagreements were resolved with a third reviewer (M.M.-O.).

### Data extraction, synthesis and effect measures

Data were independently extracted by two reviewers (F.P.-F., S.S.) and cross-checked for accuracy. Disagreements were resolved by consensus. Corresponding authors of four retrieved articles were contacted to obtain additional data regarding predictive performance, however, only one was able to provide the requested information [23]. Information extracted included: publication (author, year, sample size, country of origin, study cohort and study design), participants (age, sex), proportion of participants stratified in each risk category, and falls rate for each risk strata. Potential conflict of interests declared or not declared were also examined. A customised Excel spreadsheet was used for screening and data extraction. Data synthesis and effect measures included the distribution of participants across WFG risk categories, diagnostic accuracy (sensitivity, specificity, accuracy) and fall rates for each risk stratification group.

Statistical values were extracted directly from the included articles. Sensitivity, specificity and accuracy reported in the studies were calculated by comparing fall-risk stratification groups with fall occurrence during follow-up. Participants were classified as true positives (TP), false positives (FP), true negatives (TN) or false negatives (FN). Sensitivity was calculated as TP/(TP + FN), specificity as TN/(TN + FP), and accuracy as (TP + TN)/(TP + TN + FP + FN) [24].

Incident rate ratios (IRRs) and odds ratios (ORs) were calculated relative to the low-risk group defined by the WFG algorithm. IRRs compared fall rates between groups, whereas ORs estimated the odds of a binary outcome. Values >1 indicated greater fall risk relative to the reference group. Differences exceeding 10% were considered clinically meaningful [25, 26]. Due to heterogeneity in algorithm implementation across cohorts and the limited number of studies, findings were synthesised narratively without meta-analysis.

### Risk of bias assessment

Quality Assessment of Diagnostic Accuracy Studies version 2 (QUADAS-2) was applied to determine potential risk of bias in the included studies [27, 28]. Biases were judged as low, high or unclear across four domains: patient selection, index test performed, reference standard and flow and timing. The Cochrane framework [29] was adopted to define the reference standard for assessing prospective falls in each study including fall calendars (weekly/monthly) and structured telephone follow-up (6–12 months). Operationalised WFG algorithms with missing or incompatible tests were assigned a ‘high’ risk of bias rating. Applicability concerns were evaluated for the first three domains (patient selection, index test and reference standard) by assessing the extent to which each study matched the review question in terms of population, conduct and interpretation of the index test, and definition of the target condition. Applicability was rated as ‘low’, ‘high’ or ‘unclear’ in accordance with QUADAS-2 guidance. Two reviewers (F.P.-F., S.S.) independently assessed study quality, and disagreements were resolved through discussion with a third reviewer (M.M.-O.).

## Results

### Study selection and characteristics

The updated search conducted via the Ovid platform identified 129 records (49 from MEDLINE/PubMed and 80 from Embase). After removal of duplicates, 86 studies were retained of which 29 full texts were assessed for eligibility (Figure 2). Eight studies met the inclusion criteria, summing 25 027 participants, mean age ranged from 61 to 82 years, total female participants ranged from 42.8% to 57.8%, with a follow-up ranging from 6-months to 9 years (Table 1).

**Table 1:** Study characteristics.

Study	Study design	*N*	Mean (SD) or Median [IQR] age at baseline	% Female	Follow-up	% of ≥1 falls during follow-up	WFG algorithm operationalisation and modification
van de Loo *et al.*, Netherlands [30]	Longitudinal Aging Study Amsterdam (LASA) Population-based prospective cohort study: assessed after 1 year from baseline. Fall definition: ‘An unintentional change in position resulting in coming to rest at a lower level or on the ground’. Falls Ascertainment: falls calendars	1509	75.5 [70.1, 81.5]	52.6%	1 year	33.9%	Operationalisation: Three operationalisations were made: (i) 3KQ and Fried’s frailty criteria (ii) 3KQ and CFS classification tree (iii) Fall in the past 12 months and Fried’s frailty criteria Mobility test used: gait speed Modification made: ‘lying on the floor/unable to get up’ and ‘loss of consciousness/suspected syncope’ were excluded due to missing information For 3KQ, unsteadiness when standing or walking was assessed with tandem test as subjective unsteadiness was not available Used adjusted phrasing for 3KQ
Hicks *et al.*, Australia [23]	Sydney Memory and Ageing Study (Sydney MAS) Population-based prospective cohort study: assessed after 1 year from baseline Fall definition: ‘An unexpected event in which the person comes to rest on the ground, floor or lower level’. Falls ascertainment: returned monthly calendars	693	78.7 (4.6)	78.7%	1 year	20.8%	Operationalisation: The original algorithm and a modified low-threshold algorithm for mobility impairment were implemented. Both algorithms used 3KQ and TUG Mobility test used: TUG over the preferred gait speed Modification made: (i) Original algorithm: Used adjusted phrases for 3KQ (ii) A modified low-threshold algorithm for mobility impairment: Used adjusted phrasing for 3KQ Used TUG cut- off >10s rather than >15 s
Hartley *et al.*, Ireland [31]	Irish Longitudinal Study on Ageing (TILDA) Population-based prospective cohort study: assessed after ~2 years from Wave 1 to wave 2 Fall definition: does not explicitly state a formal fall definition. Falls ascertainment: self-reporting	5882	63.2 (9.3)	54.1%	2 years	27.5%	Operationalisation: Used 3KQ and TUG. Mobility test used: TUG over the preferred gait speed Modification made: For falls severity assessment, ‘lying on the floor/unable to get up’ was excluded due to missing information Slightly modified phrasing was used for 3KQ for unsteadiness and worries about falling
Lee *et al.*, Malaysia [32]	Malaysian Elders Longitudinal Research subset of the Transforming Cognitive Frailty into Late-Life Self-Sufficiency cohort study (AGELESS-MeLoR) Population-based prospective cohort study: assessed 2, 5 and 9 years from Wave 1 Fall definition: as ‘unintentionally coming to rest on the ground, floor, or other lower level’. Falls ascertainment: Retrospective recall of falls was used for all waves	1548	70.5^a^	42.8%	2 to 9 years	Wave 2: 20.4% Wave 3: 21.8% Wave 4: 27.0%	Operationalisation: Used 3KQ and TUG. Mobility test used: TUG over the preferred gait speed Modification made: For falls severity assessment, ‘lying on the floor/unable to get up’ was excluded due to missing information Slightly modified phrasing was used for 3KQ for unsteadiness and worries about falling
Ragusa *et al.*, USA [33]	Osteoarthritis Initiative cohort study (OAI) Prospective cohort study of individuals at risk for or with knee osteoarthritis: assessed after 2 years from baseline Fall definition: ‘an event which resulted in a person coming to rest inadvertently on the ground or floor or other lower level’. Falls ascertainment: Self-reporting-questionnaire	3981	61.4 (9.1)	58.0%	2 years	31.3%	Operationalisation: Used history of falls during the past year to assess future falls and gait speed to assess gait and balance impairment. Mobility test used: Gait speed Modification made: For falls severity assessment, ‘lying on the floor/unable to get up’ and ‘loss of consciousness/suspected syncope’ were excluded due to missing information
Li *et al.*, China [34]	China Health and Retirement Longitudinal Study (CHARLS) Population-based prospective cohort study: assessed after 2, 3, 4, 5, 7 and 9 years from baseline. Fall definition: Not provided Falls ascertainment: self-reporting-questionnaire.	9735	67.3^a^	55.0%	2 to 9 years	2-year: 21.8% 3-year: 24.6% 4-year: 36.8% 5-year: 39.1% 7-year: 50.3% 9-year: 59.7%	Operationalisation: Used history of falls during the past 2 years to assess future falls and gait speed to assess gait and balance impairment. Mobility test used: Gait speed Modification made: For the sensitive screening question, falls in the past 2 years was used instead of the past 1 year For falls severity assessment, ‘≥2 falls last year’, ‘lying on the floor/unable to get up’ and ‘loss of consciousness/suspected syncope’ were excluded due to missing information
Saunders *et al.*, Canada [35]	Cohort Study of local community-dwelling older adults-No specific cohort name assigned-prospectively assessed for falls at 1 year from baseline Fall definition: ‘an event which resulted the participant come to rest on the ground, floor or lower level’. Falls ascertainment: monthly fall diary	508	76.4	64%	1 year	52%	Operationalisation: Frailty was operationalized either by a Pictorial Fit Frailty Scale score ≥0.25 or a Clinical Frailty Scale score ≥4. Preclinical mobility limitation was assessed using the self-assessment tool. Low risk classified by excluding the following: (i) an injury resulting from a fall, (ii) two or more falls in the past year or (iii) frailty. Information about loss of consciousness, syncope, time to get off the ground after a fall, and fractures was not collected. Mobility test used: Gait speed, TUG usual pace, TUG fast pace, Brief BESTest, 5TSTS, SLS. Modification made: Added new mobility tests not originally proposed. Only 30% of participants had gait speed tested in the study.
García-Martínez *et al.*, France-Spain [36]	Falls Evaluation and Rehabilitation (FALL-ER). Participants assessed at the Emergency department after a fall with 6 months follow-up. Fall definition: ‘Any event that precipitates the patient to the ground against his/her will’ Falls ascertainment: contact after ER visit (typically by telephone follow-up).	1171	80	69.4	6 months	11.4%	Operationalisation: Barthel Index for functionality and TUG with a ≥10s cut-off. Mobility test used: none Modification: TUG was used for low-risk risk stratification whenever the patient did not report history of multiple falls and/or fracture(s). This was used for screening/stratification instead of the 3KQs. Frailty status was not assessed.

Note. CFS: Clinical Frailty Scale; TUG: Timed Up and Go test (performed at a comfortable walking speed or fast pace, as noted); TUGcog: Timed Up and Go test performed while counting backwards by 3; Brief BESTest: Brief Balance Evaluation Systems Test; 5TSTS: Five Times Sit-to-Stand test; SLS: Single-Leg Stance test.

^a^Mean age was estimated by averaging the median ages reported for each risk group.

Seven studies were secondary analyses of prospective population cohorts [23, 30–35] and one was a clinical cohort [36]. All studies evaluated risk stratification and predictive performance, except Hicks *et al.* [23] which evaluated stratification only. Van de Loo *et al.* [30] compared the WFG algorithm with the American Geriatric Society/British Geriatric Society/American Academy of Orthopaedic Surgeons algorithm and the 3KQ alone. Hartley *et al.* [31] examined feasibility and predictive utility, while Lee *et al.* [32] and Li *et al.* [34] evaluated long-term (≥2 years) predictive performance in a low- and middle-income country setting. Hicks *et al.* [23] compared stratification of falls risk and falls rates using the original WFG algorithm and a modified algorithm with a lowered TUG threshold (from 15 to 10 s). Since predictive performance was not presented in the Hicks *et al.* [23], we contacted the authors to obtain these data.

### Risk of bias

**Figure 3:**
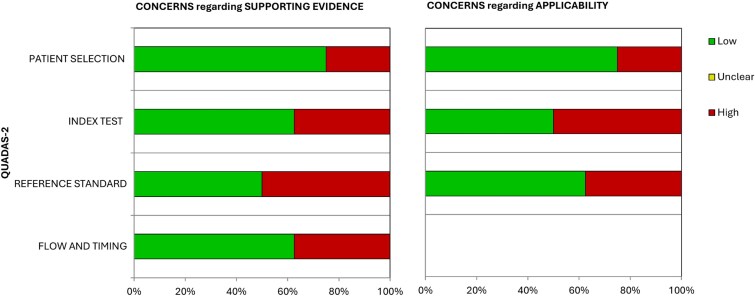
Summary of risk of bias and applicability concerns (QUADAS-2). QUADAS, Quality Assessment of Diagnostic Accuracy Studies.

Figure 3 and Supplementary Appendix 3 summarise risk of bias. Six of the eight studies were determined to have a low risk of bias for patient selection. Ragusa *et al.* [33], was rated as high risk of bias due to concerns related to participant selection, i.e. inclusion of only older people with osteoarthritis; and Garcia-Martinez *et al.* [36] also had a high risk of bias because participants were selected from an emergency department cohort. For the index test domain, low risk of bias was observed in all studies, except Ragusa *et al.* [33], Saunders *et al.* [35] and Garcia-Martinez *et al.* [36].

The reference standard domain had the highest overall risk of bias. Only four studies [23, 30, 35, 36] were assessed as low risk, whereas four studies [31–34] were judged to have a high risk of bias, due to concerns regarding the validity or independent interpretation of the reference standard. Flow and timing domain was rated as low risk in five studies but as high-risk in Hartley *et al.* [31], Lee *et al.* [32] and Garcia-Martinez *et al.* [36].

Applicability of patient selection was rated as low concern in all studies, except Ragusa *et al.* [33], which was judged as high concern because it targeted individuals at high risk of osteoarthritis rather than the general population; and Garcia-Martinez *et al.* [36] because all participants were from an emergency department cohort seeking medical help because of a previous fall. Five studies [30, 32–35] had high applicability concerns for the index test due to deviations from its intended clinical or research use, while three studies [23, 31, 36] showed low concern in this domain. For the reference standard, applicability concerns were low in five studies, but high in Hartley *et al.* [31], Ragusa *et al.* [33] and Garcia-Martinez *et al.* [36] due to misalignment between the reference standard used and the review question. Overall, the patient selection and index test domains were generally judged to be at low risk of bias, whereas the reference standard domain consistently represented the greatest source of methodological concern. Issues related to flow and timing were identified in a minority of studies. Applicability concerns were most frequently observed in the index test domain.

### Algorithms operationalisation and adaptations used

All studies assessed future falls risk using either a single question fall history [23, 30, 31, 33] or 3KQ [23, 31, 32, 34], as recommended by the WFG and one study used both measures to compare the algorithm’s predictive performance [30]. While the WFG algorithm recommended a single question of fall history within the past year, Li *et al.* [34] included a question about falls within the past 2 years. All studies using 3KQ modified the wording [23, 30–32]. In van de Loo *et al.* [30], unsteadiness when walking or standing was assessed using an objective test (tandem stance), as subjective unsteadiness data was not available [30]. For gait and balance assessment, five studies used gait speed [30, 32–35], while the other three studies used TUG as an alternative [23, 31, 36]. For the falls severity assessment, a question regarding inability to rise from the floor was included only in Hicks *et al.* [23] and Garcia-Martinez *et al.* [36]. Similarly, loss of consciousness or suspected syncope was not assessed in four studies [30, 33–35]. In Li *et al.* [34] the item ≥2 falls last year was also not assessed. van de Loo *et al.* [30] additionally included sensory assessments and alternative frailty definitions to operationalise ‘severe falls’.

### Risk stratification using the original and modified WFG algorithms

Table 2 summarises risk stratification and falls prediction for the eight selected studies. Overall, the percentage of participants classified as low risk ranged 7.9% [36] to 82.6% [34], for intermediate risk ranged from **≤1%** [23, 31, 33] up to 10.6% [23, 30, 32], while high-risk ranged from 10.5% [33] to 88.7% [36].

**Table 2:** Summary of percentage of falls risk stratification and predictive performance of the falls risk groups.

	Falls risk category and incident falls	Reported sensitivity	Reported specificity	Reported accuracy	IRR or OR (low as reference)		Main findings
					Intermediate	High	
Van de Loo *et al.* [30], Netherlands	**% of participants** *3KQ + Fried’s* Low: 61.3% Intermediate: 10.6% High: 28.1% *Single fall history question + Fried’s* Low: 78.4% Intermediate: 3.0% High: 18.6% **% of incident falls in 1 year** *3KQ + Fried’s* Low: 28.0% Intermediate: 25.9% High: 50.0% *Single fall history question + Fried’s* Low: 28.3% Intermediate: 36.6% High: 57.2%	**3KQ + Fried’s** *Low vs. High* Falls: 43.5% (40.7, 46.3) *Low vs. Intermediate-High* Falls: 48.0% (45.3, 50.7) *Low-Intermediate vs. High* Falls: 40.0% (37.4, 42.6) **3KQ + CFS** *Low vs. High* Falls: 42.2% (39.4, 45.0) *Low vs. Intermediate-High* Falls: 48.5% (45.8, 51.2) *Low-Intermediate vs. High* Falls: 37.6% (35.0,40.2) **Single fall history question + Fried’s** *Low vs. High* Falls: 31.9% (29.4, 34.4) *Low vs. Intermediate-High* Falls: 34.1% (31.6, 36.6) *Low-Intermediate vs. High* Falls: 30.9% (28.5, 33.3)	**3KQ + Fried’s** *Low vs. High* Falls: 77.0% (74.6, 79.4) *Low vs. Intermediate-High* Falls: 68.0% (65.5, 70.5) *Low-Intermediate vs. High* Falls: 79.6% (77.4, 81.8) **3KQ + CFS** *Low vs. High* Falls: 80.4% (78.1, 82.7) *Low vs. Intermediate-High* Falls: 67.7% (65.2, 70.2) *Low-Intermediate vs. High* Falls: 83.5% (81.5, 85.5) **Single fall history question + Fried’s** *Low vs. High* Falls: 87.8% (86.0, 89.6) *Low vs. Intermediate-High* Falls: 85.3% (83.4, 87.2) *Low-Intermediate vs. High* Falls: 88.2% (86.5, 89.9)	**3KQ + Fried’s** *Low vs. High* Falls: 65.4% (62.7, 68.1) *Low vs. Intermediate-High* Falls: 61.2% (58.6, 63.8) *Low-Intermediate vs. High* Falls: 66.3% (63.8, 68.8) **3KQ + CFS** *Low vs. High* Falls: 66.9% (64.2, 69.6) *Low vs. Intermediate-High* Falls: 61.2% (58.6, 63.8) *Low-Intermediate vs. High* Falls: 67.9% (65.4, 70.4) **Single fall history question + Fried’s** *Low vs. High* Falls: 69.0% (66.5, 71.5) *Low vs. Intermediate-High* Falls: 68.0% (65.5, 70.5) *Low-Intermediate vs. High* Falls: 68.8% (66.3, 71.3)	IRR **3KQ + Fried’s** Falls: 0.93 Fracture: 2.58 **Single fall history question + Fried’s** Falls: 1.29 Fractures: 2.58	IRR **3KQ + Fried’s** Falls: 1.79 Fractures: 2.16 **Single fall history question + Fried’s** Falls: 2.02 Fractures: 2.16	Confirmed that 3KQ increased sensitivity over single-item fall history Both frailty scales are comparable in sensitivity and add sensitivity over single fall history question
Hicks *et al.* [23], Australia	**% of participants** *Original algorithm* Low: 68.0% Intermediate: 0.7% High: 31.3% *Modified algorithm* Low: 50.4% Intermediate: 18.3% High: 31.3% **% of fallers in 1 year** *Modified algorithm* Low: 35.8% Intermediate: 32.3% High: 63.1%	**Original algorithm** *Low vs. High* Falls: 45.4% (39.8–51.0)^a^ **Modified algorithm** *Low vs. High* Falls: 52.3% (46.2–58.3)^a^ *Low vs. Intermediate* Falls: 24.7% (18.6–31.6)^a^ *Intermediate vs. High* Falls: 77.0% (70.4–82.7)^a^	**Original algorithm** *Low vs. High* Falls: 79.1% (74.8–82.9)^a^ **Modified algorithm** *Low vs. High* Falls: 73.4% (68.2–78.2)^a^ *Low vs. Intermediate* Falls: 72.2% (67.0–77.0)^a^ *Intermediate vs. High* Falls: 51.5% (43.9–59.1)^a^	NA	IRR **Original algorithm** Falls: 0.33 (0.04–3.09) **Modified algorithm:** Falls: 1.22 (0.86–1.73)	IRR **Original algorithm** Falls: 2.35 (1.96–2.78) **Modified algorithm** Falls: 2.49 (1.96–3.16)	Original algorithm has low discrimination for the intermediate group The two modifications proposed provided a better stratification into three sizable fall risk groups. The intermediate risk group is well represented and may be at risk of transitioning to high fall rates in the medium to long term
Hartley *et al.* [31], Ireland	**% of participants** Low: 77% Intermediate: 1% High: 22% **% of incident falls in 2 years** Low: 20.1% Intermediate: 44.8% High: 45.4%	*Low vs. High* Falls: 47.4% *Low vs. Intermediate-High* Falls: 48.5%	*Low vs. High* Falls: 78.6% *Low vs. Intermediate-High* Falls: 77.8%	*Low vs. High* Falls: 70.1% *Low vs. Intermediate-High* Falls: 69.8%	IRR Falls: 2.22	IRR Falls: 2.25	High specificity for falls prediction Low discrimination for intermediate risk group 60% of low-risk individuals suffered injuries after fall
Lee *et al.* [32], Malaysia	**% of participants** *Wave 1 (baseline)* Low: 74.6% Intermediate: 6.4% High: 19.0% *Wave 2* Low: 73.7% Intermediate: 6.4% High: 19.9% *Wave 3* Low: 79.4% Intermediate: 5.5% High: 15.2% *Wave 4* Low: 77.2% Intermediate: 6.3% High: 16.4% **% of incident falls in 2, 5 and 9 years** *Wave 1 (baseline)* Low: 6.1% Intermediate: 13.1% High: 86.4% *Wave 2* Low: *13.4*% Intermediate: 29.8% High: 42.9% *Wave 3* Low: 19.4% Intermediate: 25.5% High: 32.8% *Wave 4* Low: 25.8% Intermediate: 27.7% High: 27.0%	*Low vs. Intermediate-High* *Wave 2* Falls: 51.3% (43.1–59.2) *Wave 3* Falls: 29.4% (23.1–36.6) *Wave 4* Falls: 26.0% (20.2–32.8)	*Low vs. Intermediate-High* *Wave 2* Falls: 80.1% (76.6–83.2) *Wave 3* Falls: 81.6% (78.5–84.5) *Wave 4* Falls: 78.4% (74.7–81.8)	*Low vs. Intermediate-High* *Wave 2* Falls: 74.2% (71.1–77.4) *Wave 3* Falls: 70.3% (67.2–73.3) *Wave 4* Falls: 64.3% (60.8–67.7)	IRR *Wave 1 (baseline)* Falls: 2.14 *Wave 2* Falls: 2.22 Fractures: 2.26 *Wave 3* Falls: 1.31 Fractures: 2.41 *Wave 4* Falls: 1.07 Fractures: 2.28	IRR *Wave 1 (baseline)* Falls: 14.1 *Wave 2* Falls: 3.20 Fractures: 0.91 *Wave 3* Falls: 1.69 Fractures: 0.82 *Wave 4* Falls: 1.04 Fractures: 0.94	Moderate predictive ability for falls Predictive strength fades out over time in following study waves
Ragusa *et al.* [33], USA	**% of participants** Low: 46.9% Intermediate: 0.42% High: 10.5% **% of incident falls in 2 years** Low: 25.1% Intermediate: 36% High: 60.6%	*Low vs. High* Falls: 33.76% *Low vs. Intermediate-High* Falls: 89.99%	*Low vs. High* Falls: 34.24% *Low vs. Intermediate-High* Falls: 89.47%	*Low vs. High* Falls: 72.4% *Low vs. Intermediate-High* Falls: 72.1%	IRR Falls: 1.43 Injury: 0 (no injuries)	IRR Falls: 2.41 Injury: 6.12	WFG modified assessment algorithm distinguished those at greater risk of falling using the opportunistic case finding method Good specificity, but limited sensitivity of falls risk stratification algorithm.
Li *et al*. [34], China	**% of participants** *Year 2* Low: 82.6% Intermediate: 3.5% High: 13.9% *Year 3* Low: 83.2% Intermediate: 4.2% High: 12.6% *Year 4* Low: 81.5% Intermediate: 3.0% High: 15.5% *Year 5* Low: 83.1% Intermediate: 4.1% High: 12.8% *Year 7* Low: 82.0% Intermediate: 3.5% High: 14.5% *Year 9* Low: 80.4% Intermediate: 3.1% High: 16.5% **Falls incidence % in 2, 3, 4, 5, 7 and 9 years** *Year 2* Low: 18.3% Intermediate: 35.2% High: 39.2% *Year 3* Low: 20.9% Intermediate: 36.7% High: 45.2% *Year 4* Low: 32.4% Intermediate: 54.9% High: 56.6% *Year 5* Low: 34.5% Intermediate: 59.8% High: 62.4% *Year 7* Low: 46.1% Intermediate: 67.6% High: 69.9% *Year 9* Low: 55.54% Intermediate: 72.1% High: 78.2%	*Low vs. High* *Year 2* Falls: 26.5% (24.6, 28.5), Injury: 30.1% (27.2, 33.2) *Year 3* Falls: 24.6% (22.2, 27.1), Injury: 28.0% (24.3, 31.9) *Year 4* Falls: 24.9% (22.6, 27.2), Injury: 27.3% (24.1, 30.6) *Year 5* Falls: 21.8%(20.4, 23.2), Injury: 25.2% (23.2, 27.2) *Year 7* Falls: 21.2% (19.9, 22.6), Injury: 24.8% (23.0, 26.7), *Year 9* Falls: 22.5% (20.6, 24.4), Injury: 25.1% (22.7, 27.6) *Low-Intermediate vs. High* *Year 2* Falls: 30.6% (28.7, 32.6), Injury: 32.5% (29.5, 35.6) *Year 3* Falls: 29.5% (27.0, 32.0), Injury: 31.8% (28.1, 35.7) *Year 4* Falls: 28.2% (25.9, 30.5), Injury: 29.9% (26.7, 33.3) *Year 5* Falls: 26.6% (25.2, 28.1), Injury: 28.9% (26.9, 31.0) *Year 7* Falls: 24.9% (23.6, 26.3), Injury: 27.8% (26.0, 29.7) *Year 9* Falls: 25.4% (23.5, 27.3), Injury: 27.6% (25.2, 30.2) *Low vs. Intermediate-High* *Year 2* Fall: 25.0% (23.2, 26.9), Injury: 29.1% (26.2, 32.0) *Year 3* Fall: 23.0% (20.8, 25.4), Injury: 26.5% (23.0, 30.2) *Year 4* Fall: 23.8% (21.6, 26.0), Injury: 26.3% (23.3, 29.6) *Year 5* Fall: 20.4% (19.1, 21.7), Injury: 23.9% (22.0, 25.9) *Year 7* Fall: 20.2% (19.0, 21.5), Injury: 23.9% (22.1, 25.7) *Year 9* Fall: 21.6% (19.8, 23.5), Injury: 24.2% (21.9, 26.7)	*Low vs. High* *Year 2* Falls: 88.9% (88.1, 89.6), Injury: 87.3% (86.6, 88.0) *Year 3* Falls: 90.5% (89.6, 91.4), Injury: 88.7% (87.8, 89.6) *Year 4* Falls: 89.1% (87.9, 90.3), Injury: 86.7% (85.5, 87.9) *Year 5* Falls: 91.9% (91.1, 92.6), Injury: 89.6% (88.8, 90.3) *Year 7* Falls: 91.0% (90.0, 91.9), Injury: 89.0% (88.1, 89.8) *Year 9* Falls: 90.9% (89.2, 92.3), Injury: 88.0% (86.5, 89.4) *Low-Intermediate vs. High* *Year 2* Fall: 86.3% (85.5, 87.1), Injury: 84.3% (83.5, 85.1) *Year 3* Fall: 87.4% (86.4, 88.4), Injury: 85.2% (84.1, 86.2) *Year 4* Fall: 87.2% (85.9, 88.5), Injury: 84.2% (83.0, 85.5) *Year 5* Fall: 89.4% (88.6, 90.2), Injury: 86.1% (85.3, 86.9) *Year 7* Fall: 88.9% (87.9, 89.9), Injury: 86.0% (85.1, 86.9) *Year 9* Fall: 88.9% (87.1, 90.5), Injury: 85.4% (83.8, 86.9) *Low vs. Intermediate-High* *Year 2* Fall: 89.2% (88.5, 89.9), Injury: 87.8% (87.1, 88.5) *Year 3* Fall: 90.9% (89.9, 91.7), Injury: 89.2% (88.3, 90.1) *Year 4* Fall: 89.4% (91.4, 92.8), Injury: 87.1% (85.9, 88.2) *Year 5* Fall: 92.1% (90.3, 92.1), Injury: 90.0% (89.3, 90.6) *Year 7* Fall: 91.2% (90.3, 92.1), Injury: 89.4% (88.5, 90.2) *Year 9* Fall: 91.0% (89.4, 92.5), Injury: 88.3% (86.9, 89.7)	NA	OR *Year 2* Falls: 2.36 (1.87, 2.97) Injury: 1.2 *Year 3* Falls: 2.14 (1.61, 2.84) Injury: 1.47 (0.99, 2.18) *Year 4* Falls: 2.50 (1.73, 3.62) Injury: 1.49 (0.96, 2.30) *Year 5* Falls: 2.62 (2.12, 3.24) Injury: 1.44 (1.13, 1.84) *Year 7* Falls: 2.36 (1.81, 3.07) Injury: 1.35 (1.03, 1.75) *Year 9* Falls: 2.01 (1.29, 3.14) Injury: 1.34 (0.89, 2.02)	OR *Year 2* Falls: 2.44 (2.15, 2.78) Injury: 2.56 *Year 3* Falls: 2.57 (2.16, 3.06) Injury: 2.49 (2.01, 3.09) *Year 4* Falls: 2.32 (1.94, 2.77) Injury: 2.14 (1.75, 2.60) *Year 5* Falls: 2.55 (2.24, 2.90) Injury: 3.40 (2.09, 2.75) *Year 7* Falls: 2.23 (1.93, 2.57) Injury: 2.24 (1.95, 2.56) *Year 9* Falls: 2.41 (1.93, 3.01) Injury: 2.09 (1.72, 2.54)	WFG algorithm is valid for identifying fall risk categories but insufficient alone for predicting future falls or injuries in Chinese community-dwelling older adults Sensitivity improves after the 5 years of follow-up while specificity declines
Saunders *et al.* [35], Canada	**% of participants** Low: 62.2% Intermediate: 9% High: 28.7% **% of incident falls in 1 year** Low: 17.3% Intermediate: 20.2% High: 62% **% of incident injurious falls in 1 year** Low: 39.8% Intermediate: 40% High: 27.8%	NA	NA	NA	IRR: Falls: 1.11 (0.79–1.57) Injury: NA	IRR: Falls: 2.64 (1.96–3.56) Injury: NA	Adding balance and mobility tests do not provide additional discriminatory value within the current WFG algorithm. Gait speed was the best predictor of future falls with (<0.78 m/s) and without injuries (<1.21 m/s).
García-Martínez *et al.* [36], France-Spain	**% of participants** Low: 7.9% Intermediate: 3.3% High: 88.7% **% of incident falls in 6 months** Low: 7.5% Intermediate: 15.4% High: 11.6% **% of incident injurious falls in 6 months** Low: 39.8% Intermediate: 40% High: 27.8%	Low vs High: 90% (85%–95%)	Low vs High: 11% (10%–13%)	NA	OR: Falls: 1.98 (0.61–6.46) Injury: NA	OR: Falls: 1.53 (0.68–3.43) Injury: NA	WFG algorithm risk stratification had high sensitivity to identify older individuals in the Emergency Department that would have a new fall 6 months after their discharge.

^a^Data provided by the corresponding author; NA, data is not available for reporting.

Only two studies compared the WFG original algorithm with their modified versions for risk stratification. Specifically, in the van de Loo *et al.* [30], the intermediate risk group increased from 3.0% using falls history to 10.6% when using 3KQ instead. In Hicks *et al.* [23], the intermediate risk group increased from 0.7% to 18.3% following a lowered TUG threshold.

### Falls and fractures incident risk and discrimination

Across studies (Table 2), fall rates increased with higher WFG fall risk categories. In low-risk groups, fall incidences ranged from 17.3% to 35.8% at 1 year [23, 35], and 13.4% to 25.1% at 2 years [32, 33]. In intermediate-risk groups, the incidence of falls was between 20.2% and 36.6% at 1 year [23, 30, 35], and between 13.1% and 44.8% in 2 years [31, 32]. High-risk groups had the highest fall incidence, with 1-year incidences of 50% to 63.1% [23, 30] and 2-year incidence of 39.2% to 86.4% [32, 34].

While IRRs for high-risk participants were generally higher than for intermediate-risk participants at 1- or 2-years follow-up, Hartley *et al.* [31] found comparable risks, with IRRs of 2.22 for intermediate-risk and 2.25 for high-risk. Intermediate-risk groups demonstrated modest increases in fall incidence compared with low-risk, with IRRs ranging from 1.11 [35] to 1.29 [30] over 1 year, 1.43 [33] to 2.36 [34] over 2 years and 1.34 over 9 years [34]. High-risk groups consistently showed higher fall rates, with IRRs ranging from 1.79 [30] to 2.49 for 1 year, and from 2.25 [31] to 3.20 [32] for over 2 years [31–34]. Elevated falls risk persisted in the intermediate- and high-risk groups during longer follow-up periods (>2 years), in both Li *et al.* [34] and Lee et al [32]. However, while the magnitude of risk remained stable over time in Li *et al.* [34], it decreased substantially in Lee *et al.* [32]. Importantly, lowering the proposed TUG cut-off by the WFG increased the IRR for the intermediate-risk group from 0.33 (original threshold) to 1.22 (reduced threshold) [23].

IRRs for injurious falls were reported in Ragusa *et al.* [33], Li *et al.* [34], while van de Loo *et al.* [30] and Lee *et al.* [32] reported IRRs for fractures. For fractures, IRRs were higher in intermediate-risk groups than in high-risk group (2.58 vs. 2.16 in van de Loo *et al.* [30] and 2.26 vs 0.91 in Lee *et al.* [32]). In contrast, IRRs for injurious falls were higher in high-risk groups than in intermediate-risk groups (6.12 vs. 0 in Ragusa *et al.* [33] and 2.56 vs. 1.2 in Li *et al.* [34]).

### WFG algorithm sensitivity, specificity and accuracy for predicting falls

The predictive performance for falls incidence reported in this review corresponds to the earliest follow-up time point from baseline (i.e. first follow up assessment at 6–24 months; Table 2). Six studies compared low vs. high risk [23, 30–33, 36], five compared low vs. intermediate-high risk [30–34], two studies compared low-intermediate vs. high risk [30, 34], and one study compared low vs. intermediate risk and intermediate vs. high risk [23].

Sensitivity ranged from 26.5% to 90% for low vs. high risk [23, 30, 31, 33, 34, 36], 25% to 89.99% for low vs. intermediate-high risk [30–33], and 30.6% to 40% for low-intermediate vs. high risk [30, 34]. Specificity ranged from 11% to 89.99% for low vs. high risk [30, 31, 33, 34], 68% to 89.47% for low vs. intermediate-high risk [30–34], and 79.6% to 86.3% for low-intermediate vs. high risk [30]. Accuracy ranged from 65.4% to 72.4% for low vs. high risk [30, 31, 33], and 61.2% vs. 74.2% for low vs. intermediate-high risk [30, 31, 33]. Accuracy for predicting falls incidence for low-intermediate vs. high risk was only evaluated in van de Loo *et al.* [30] with values of 66.3% using 3KQ and 68.8% using fall history.

Use of 3KQ yielded higher sensitivity, specificity and accuracy for all comparisons in van de Loo et al [30]. In Hicks *et al.* [23] the algorithm with the lowered TUG threshold increased sensitivity for low vs. high risk (from 45.4% using the original algorithm to 52.3%), but decreased specificity. Using the lowered TUG threshold, sensitivity was 24.7% for low vs. intermediate risk and 77.0% for intermediate vs. high risk, with corresponding specificities of 72.2% and 51.5%, respectively [23]. In the Saunders *et al.* study [35], universal gait speed assessment improved sensitivity of fallers detection by 56% and was the strongest predictor of both falls and injurious falls (AUC 0.59 and 0.70; cut-offs 1.21 and 0.78 m/s, respectively) [35]. Overall, sensitivity declined over time with longer follow-ups, whereas specificity and accuracy remained stable or increased slightly for all comparisons. A similar pattern was observed for injurious falls in Li *et al.* [34].

## Discussion

We aimed to determine whether the WFG algorithm, in its original or modified forms, prospectively stratifies community-dwelling older adults into low-, intermediate- and high-risk categories for future falls, and whether it has been prospectively validated and appropriately operationalised. All studies used pre-existing datasets, and no study applied the algorithm exactly as proposed, with two studies [23, 30] making slight changes to operationalize its use. The remaining studies adapted the algorithm for their datasets using alternative variables, including rewording the 3KQ [33, 34], using alternative frailty measures [30, 31], excluding syncope [30, 33–35] and the inability to rise from the floor items [30–32, 34, 35]. These adaptations, driven by data limitations rather than deliberate methodological changes, provide insight into the algorithm’s flexibility and real-world applicability.

Overall, the WFG algorithm consistently identified older adults at high risk of future falls with high specificity, although its sensitivity was relatively low. Risk stratification varied considerably across cohorts, likely reflecting adaptations to the algorithm that contributed to heterogeneity in findings. Most participants were classified as either low or high risk, with relatively few assigned to the intermediate-risk category. Individuals classified as high risk experienced a two- to three-fold higher incidence of falls than those classified as low risk.

There was a variation in high-risk classification across studies, partly reflecting missing information on ‘concerns about falls’, ‘unsteadiness’, ‘syncope’ or ‘inability to get up from the floor’, leading to lower reported high-risk prevalence. The variation and low prevalence of the intermediate-risk category across studies raises questions as to whether this group represents a distinct clinical phenotype or reflects differences in algorithm operationalisation, mainly related to the mobility tests and the cut-off threshold used.

The WFG algorithm previously recommended gait speed, using a conservative cut-off (≤0.8 m/s), rather than the TUG to prioritise specificity over sensitivity given the TUG’s lower predictive performance [12, 21]. Mobility testing was recommended only for individuals with a history of falls or self-reported gait or balance problems. However, recent evidence suggests that up to one-third of older adults without prior falls or self-reported mobility problems experience a fall within 1 year, and a gait speed <1.0 m/s is associated with up to a two-fold increased fall risk [19]. Similarly, the study included by Hicks *et al.* [23] found that applying a lower TUG threshold (>10 s rather than 15 s) reclassified some individuals from low to intermediate risk, suggesting that low-risk status should be assigned only after confirming normal mobility. Another two cross-sectional cohort studies also found using the 3KQ combined with a gait speed test universally increased the proportion of individuals classified as intermediate risk nearly four-fold [37, 38]. Clarifying the role of the intermediate-risk category is important, as it likely identifies individuals who would benefit from early preventive interventions focusing on balance and functional strength training.

Our findings align with the broader literature on fall-risk assessment, demonstrating a trade-off between sensitivity and specificity, with the WFG algorithm, particularly in its original form, showing good specificity but limited sensitivity. Future refinements could explore whether adding domains such as cognition, sensory deficits or environmental factors may improve sensitivity without compromising feasibility. When resources permit, 3KQ should be used rather than fall history alone as an entry question, as it improves sensitivity. Regardless of the mobility test selected, our results suggest the universal use of mobility assessment to improve risk stratification and identification of intermediate-risk groups, with cut-off selection influencing the size of identified risk strata, as illustrated in our proposed modification (Figure 1B).

Several limitations of our systematic review should be acknowledged. First, the eight included studies were heterogeneous in their operationalisation of the WFG algorithm and did not apply it as originally proposed, instead modifying it to fit available data. Prospective studies specifically designed to evaluate the WFG algorithm are needed to assess the performance of both original and modified versions and identify the most accurate and feasible mobility assessments. Second, although participants were community-dwelling older adults, limited information on cognitive status may have introduced bias in fall-risk assessment. Third, differences in fall definitions, follow-up duration and outcome ascertainment may have reduced comparability across studies. Finally, the WFG algorithm was developed for community-dwelling older adults and is unlikely to be discriminative in residential care settings, where most residents would be classified as high risk because of greater frailty, impaired mobility and higher baseline fall risk. Accordingly, the WFG guidelines recommend treating all residential care residents as high risk and prioritising comprehensive assessment and management over screening. Despite these limitations, we empirically evaluated the WFG algorithm and have proposed a modified version for further testing.

In conclusion, modifications to the WFG algorithm improved sensitivity and enhanced discrimination of intermediate-risk groups. Universal mobility testing and more strict cut-offs may increase its utility for clinical and public health fall prevention, although these benefits must be balanced against greater assessment burden. The evidence from this review supports a proposed modification to the WFG algorithm that warrants validation in prospectively designed cohort studies.

## Supplementary Material

aa-26-0315-File002_afag231

## References

[1] Tinetti ME, Speechley M, Ginter SF. Risk factors for falls among elderly persons living in the community. N Engl J Med 1988;319:1701–7. 3205267 10.1056/NEJM198812293192604

[2] Ellmers TJ, Ventre JP, Freiberger E et al. Does concern about falling predict future falls in older adults? A systematic review and meta-analysis. Age Ageing 2025;54:1–16. 10.1093/ageing/afaf089 PMC11976718 40197783

[3] Tinetti ME, Speechley M. Prevention of falls among the elderly. N Engl J Med 1989;320:1055–9. 2648154 10.1056/NEJM198904203201606

[4] Speechley M, Tinetti M. Falls and injuries in frail and vigorous community elderly persons. J Am Geriatr Soc 1991;39:46–52. 1987256 10.1111/j.1532-5415.1991.tb05905.x

[5] Sherrington C, Michaleff ZA, Fairhall N et al. Exercise to prevent falls in older adults: an updated systematic review and meta-analysis. Br J Sports Med 2017;51:1750–8. 27707740 10.1136/bjsports-2016-096547

[6] Burns E, Kakara R. Deaths from falls among persons aged >/=65 years–United States, 2007-2016. MMWR Morb Mortal Wkly Rep 2018;67:509–14. 29746456 10.15585/mmwr.mm6718a1 PMC5944976

[7] Florence CS, Bergen G, Atherly A et al. Medical costs of fatal and nonfatal falls in older adults. J Am Geriatr Soc 2018;66:693–8. 29512120 10.1111/jgs.15304 PMC6089380

[8] Parachute. The Cost of Injury in Canada. Toronto (ON): Parachute; 2015.

[9] Garnett MF, Weeks JD, Zehner AM. Unintentional fall deaths in adults age 65 and older: United States, 2023. NCHS Data Brief 2025;532:1. 10.15620/cdc/174601 PMC12869897 41329986

[10] Santos-Lozada AR . Trends in deaths from falls among adults aged 65 years or older in the US, 1999-2020. JAMA 2023;329:1605–7. 37159042 10.1001/jama.2023.3054 PMC10170339

[11] Rommers E, De Pauw R, Petrovic M et al. Epidemiology of falls in community-dwelling older adults in Europe: a systematic review and meta-analysis. Age Ageing 2025;54:1–10. 10.1093/ageing/afaf157 40479612

[12] Montero-Odasso M, van der Velde N, Martin FC et al. World guidelines for falls prevention and management for older adults: a global initiative. Age Ageing 2022;51:1–36 10.1093/ageing/afac205 PMC9523684 36178003

[13] Leung PB, Alexander JT, Ouchida KE. Falls prevention for older adults. JAMA 2024;331:1409–10. 38536162 10.1001/jama.2023.26942

[14] Seppala LJ, Frith J, Skelton DA et al. Challenges and opportunities for falls prevention: an online survey across European healthcare professionals. Eur Geriatr Med 2025;16:1269–82. 40528097 10.1007/s41999-025-01237-5 PMC12378296

[15] Seppala LJ, Lord SR, Masud T et al. The world falls guidelines: how is implementation progressing globally? Age Ageing 2025;54; 1-6. 10.1093/ageing/afaf214 40794908

[16] Peel NM, Kassulke DJ, McClure RJ. Population based study of hospitalised fall related injuries in older people. Inj Prev 2002;8:280–3. 12460962 10.1136/ip.8.4.280 PMC1756575

[17] Burns ER, Lee R, Hodge SE et al. Validation and comparison of fall screening tools for predicting future falls among older adults. Arch Gerontol Geriatr 2022;101:104713. 35526339 10.1016/j.archger.2022.104713 PMC10543920

[18] Campbell AJ, Borrie MJ, Spears GF. Risk factors for falls in a community-based prospective study of people 70 years and older. J Gerontol 1989;44:M112–7. 2738307 10.1093/geronj/44.4.m112

[19] Montero-Odasso M, Pieruccini-Faria F, Son S et al. Fall risk stratification in older adults: low and not-at-risk status still associated with falls and injuries. Age Ageing 2025;54:1–9. 10.1093/ageing/afaf064 PMC11942794 40139220

[20] Eckstrom E, Parker EM, Lambert GH et al. Implementing STEADI in academic primary care to address older adult fall risk. Innov Aging 2017;1(2):1-9. 10.1093/geroni/igx028 PMC6016394 29955671

[21] Beck Jepsen D, Robinson K, Ogliari G et al. Predicting falls in older adults: an umbrella review of instruments assessing gait, balance, and functional mobility. BMC Geriatr 2022;22:615. 35879666 10.1186/s12877-022-03271-5 PMC9310405

[22] Moher D, Altman DG, Liberati A et al. PRISMA statement. Epidemiology 2011;22:128 author reply. 21150360 10.1097/EDE.0b013e3181fe7825

[23] Hicks C, Menant J, Delbaere K et al. Two simple modifications to the World Falls Guidelines algorithm improves its ability to stratify older people into low, intermediate and high fall risk groups. Age Ageing 2024;53:1–8. 10.1093/ageing/afae192 39348910

[24] Akobeng AK . Understanding diagnostic tests 1: sensitivity, specificity and predictive values. Acta Paediatr 2007;96:338–41. 17407452 10.1111/j.1651-2227.2006.00180.x

[25] Billington J, Fahey T, Galvin R. Diagnostic accuracy of the STRATIFY clinical prediction rule for falls: a systematic review and meta-analysis. BMC Fam Pract 2012;13:76. 22870921 10.1186/1471-2296-13-76 PMC3460792

[26] Mickey RM, Greenland S. The impact of confounder selection criteria on effect estimation. Am J Epidemiol 1989;129:125–37. 2910056 10.1093/oxfordjournals.aje.a115101

[27] Whiting PF, Rutjes AW, Westwood ME et al. QUADAS-2: a revised tool for the quality assessment of diagnostic accuracy studies. Ann Intern Med 2011;155:529–36. 22007046 10.7326/0003-4819-155-8-201110180-00009

[28] Schueler S, Schuetz GM, Dewey M. The revised QUADAS-2 tool. Ann Intern Med 2012;156:323 author reply -4. 22351721 10.7326/0003-4819-156-4-201202210-00018

[29] Bossuyt PM, Deeks JJ, Leeflang MM et al. Evaluating medical tests: introducing the Cochrane Handbook for Systematic Reviews of Diagnostic Test Accuracy. Cochrane Database Syst Rev 2023;7:ED000163. 37470764 10.1002/14651858.ED000163 PMC10408284

[30] van de Loo B, Heymans MW, Medlock S et al. Retrospective evaluation of the world falls guidelines-algorithm in older adults. Age Ageing 2024;53:1–8. 10.1093/ageing/afae229 PMC11488976 39424356

[31] Hartley P, Forsyth F, Rowbotham S et al. The use of the World Guidelines for Falls Prevention and Management’s risk stratification algorithm in predicting falls in the Irish Longitudinal Study on Ageing (TILDA). Age Ageing 2023;52:1–9. 10.1093/ageing/afad129 PMC10353759 37463283

[32] Lee SJS, Tan MP, Mat S et al. Predictive value of the World falls guidelines algorithm within the AGELESS-MELoR cohort. Arch Gerontol Geriatr 2024;125:105523. 38878671 10.1016/j.archger.2024.105523

[33] Ragusa FS, Di Bella G, Dominguez LJ et al. The role of the World Guidelines for Falls Prevention and Management’s risk stratification algorithm in predicting falls: a retrospective analysis of the Osteoarthritis Initiative. Age Ageing 2024;53:1–7. 10.1093/ageing/afae187 PMC11974246 39171386

[34] Li W, Zhao M, Fu Y et al. Performance of risk assessment algorithms recommended by the World Falls Guidelines (WFG) to predict fall and fall-related injury among older Chinese community-dwellers. J Safety Res 2025;95:7. 10.1016/j.jsr.2025.10.018 41338789

[35] Saunders S, Speechley M, Griffith LE et al. Prospective evaluation of balance and mobility tests as part of the World Falls Guidelines algorithm. Age Ageing 2026;55:1–10. 10.1093/ageing/afag028 41697879

[36] García-Martínez A, Artajona L, García-Rosa S et al. Fall risk prediction in older adults at the emergency department: where the guidelines do not fit. Emerg Med J 2026; Advance online publication. 10.1136/emermed-2025-215651. 41850758

[37] de Avelar NCP, Danielewicz AL, Moreira BS et al. Fall risk assessment using the World Guidelines for Falls Prevention Algorithm: evidence from the ELSI-Brazil study. Eur Geriatr Med 2026;17(3):1-8. 10.1007/s41999-026-01446-6 PMC13309500 41917319

[38] Blain H, Bernard PL, Berrut G et al. Assessment of the “world guidelines for falls prevention and management” algorithm in older volunteers. Aging Clin Exp Res 2026; Advance online publication. 10.1007/s40520-026-03405-4 PMC13216084 42201438

