# Supplementary material for: Validation of the World Falls Guidelines risk stratification algorithm. A systematic review
Source: Age Ageing. 2026 Aug 12;55(8):afag231. doi: 10.1093/ageing/afag231 (PMC13464692; doi:10.1093/ageing/afag231)
Supplement: Supplementary file 1 [file aa-26-0315-file002_afag231.docx]

**Validation of the World Falls Guidelines Risk Stratification Algorithm. A Systematic Review.**

Contents

[Appendix 1 – PRISMA-DTA table	2](#_Toc235175415)

[Appendix 2 – Search strategy in Ovid	4](#_Toc235175416)

[Appendix 3 – Risk of bias table summary	5](#_Toc235175417)

# Appendix 1 – PRISMA-DTA table

| **PRISMA-DTA tableSection/topic** | **#** | **PRISMA-DTA Checklist Item** | **Reported on page #** |
| --- | --- | --- | --- |
| **TITLE / ABSTRACT** | | |  |
| Title | 1 | Identify the report as a systematic review (+/- meta-analysis) of diagnostic test accuracy (DTA) studies. | Title page (separate) |
| Abstract | 2 | Abstract: See PRISMA-DTA for abstracts. | Pg. 1 |
| **INTRODUCTION** | | |  |
| Rationale | 3 | Describe the rationale for the review in the context of what is already known. | Pg. 2 (introduction paragraph 1 and 2) |
| Clinical role of index test | D1 | State the scientific and clinical background, including the intended use and clinical role of the index test, and if applicable, the rationale for minimally acceptable test accuracy (or minimum difference in accuracy for comparative design). | Pg. 2-3 |
| Objectives | 4 | Provide an explicit statement of question(s) being addressed in terms of participants, index test(s), and target condition(s). | Last paragraphs pg.2-3 |
| **METHODS** | | |  |
| Protocol and registration | 5 | Indicate if a review protocol exists, if and where it can be accessed (e.g., Web address), and, if available, provide registration information including registration number. | Pg. 3 (Methods) |
| Eligibility criteria | 6 | Specify study characteristics (participants, setting, index test(s), reference standard(s), target condition(s), and study design) and report characteristics (e.g., years considered, language, publication status) used as criteria for eligibility, giving rationale. | Pg. 3 (eligibility criteria) |
| Information sources | 7 | Describe all information sources (e.g., databases with dates of coverage, contact with study authors to identify additional studies) in the search and date last searched. | Pg. 3 (search strategy) |
| Search | 8 | Present full search strategies for all electronic databases and other sources searched, including any limits used, such that they could be repeated. | Pg. 3 (search strategy) |
| Study selection | 9 | State the process for selecting studies (i.e., screening, eligibility, included in systematic review, and, if applicable, included in the meta-analysis). | Pg. 3 (search strategy and study selection) |
| Data collection process | 10 | Describe method of data extraction from reports (e.g., piloted forms, independently, in duplicate) and any processes for obtaining and confirming data from investigators. | Pg. 3-4 (Data extraction) |
| Definitions for data extraction | 11 | Provide definitions used in data extraction and classifications of target condition(s), index test(s), reference standard(s) and other characteristics (e.g. study design, clinical setting). | Pg. 4 (Data extraction) |
| Risk of bias and applicability | 12 | Describe methods used for assessing risk of bias in individual studies and concerns regarding the applicability to the review question. | Pg. 4 (Risk of bias Assessment) |
| Diagnostic accuracy measures | 13 | State the principal diagnostic accuracy measure(s) reported (e.g. sensitivity, specificity) and state the unit of assessment (e.g. per-patient, per-lesion). | Pg. 4 (Effect Measures and Data Synthesis) |
| Synthesis of results | 14 | Describe methods of handling data, combining results of studies and describing variability between studies. This could include, but is not limited to: a) handling of multiple definitions of target condition. b) handling of multiple thresholds of test positivity, c) handling multiple index test readers, d) handling of indeterminate test results, e) grouping and comparing tests, f) handling of different reference standards | Pg. 4-5 (Effect Measures and Data Synthesis) |

# Appendix 2 – Search strategy in Ovid

Ovid search using MEDLINE and EMBASE databases:

| **Search** | **terms** | **Results** |
| --- | --- | --- |
| **1** | **(world falls guidelines or world guidelines).mp. [mp=ti, ab, hw, tn, ot, dm, mf, dv, kf, fx, dq, bt, nm, ox, px, rx, ui, sy, ux, mx]** | 193 |
| **2** | **(falls or accidental falls).mp. [mp=ti, ab, hw, tn, ot, dm, mf, dv, kf, fx, dq, bt, nm, ox, px, rx, ui, sy, ux, mx]** | 190625 |
| **3** | **1 and 2** | 129 |

“world falls guidelines" OR "World guidelines" AND "falls" OR "accidental falls” searching for these fields simultaneously, not just titles/abstracts including: ti = Title; ab = Abstract; hw = Heading word (words from subject headings); tn = Trade name; ot = Original title; dm = Device manufacturer; mf = Manufacturer; dv = Device; kf = Keyword heading word; fx = Floating subheading word; dq = Drug qualifier; bt = Book title; nm = Name of substance; ox = Other term; px = Pharmacologic action; rx = Registry word; ui = Unique identifier; sy = Synonym; ux = Unknown field / supplementary concept; mx = Miscellaneous field"

# Appendix 3 – Risk of bias table summary

**Supplementary** **Table 1- Summary of Risk of Bias and Applicability Concerns (QUADAS-2).**

| **Study** | **Supporting evidence**  **(QUADAS-2)** | | | |  | **Applicability concerns**  **(QUADAS-2)** | | |
| --- | --- | --- | --- | --- | --- | --- | --- | --- |
|  | **P** | **I** | **R** | **FT** |  | **P** | **I** | **R** |
| van de Loo et al., 2024 [32] | ✓ | ✓ | ✓ | ✓ |  | ✓ | ✗ | ✓ |
| Hartley et al., 2023 [35] | ✓ | ✓ | ✗ | ✗ |  | ✓ | ✓ | ✗ |
| Lee et al., 2024 [31] | ✓ | ✓ | ✗ | ✗ |  | ✓ | ✗ | ✓ |
| Hicks et al., 2024 [23] | ✓ | ✓ | ✓ | ✓ |  | ✓ | ✓ | ✓ |
| Ragusa et al., 2024 [33] | ✗ | ✗ | ✗ | ✓ |  | ✗ | ✗ | ✗ |
| Li et al (2025) [30] | ✓ | ✓ | ✗ | ✓ |  | ✓ | ✗ | ✓ |
| Saunders et al., 2026 [34] | ✓ | ✗ | ✓ | ✓ |  | ✓ | ✗ | ✓ |
| Garcia-Martinez et al., 2026 [36] | ✗ | ✗ | ✓ | ✗ |  | ✗ | ✓ | ✗ |

P = patient selection; I = index test; R = reference standard; FT = flow and timing. ✓ indicates low risk; ✗ indicates high risk; ? indicates unclear risk.
